# Symptom Perceptions and Help-Seeking Behaviours of Omani Patients Diagnosed with Late-Stage Colorectal Cancer: A Qualitative Study

**DOI:** 10.31557/APJCP.2021.22.2.427

**Published:** 2021-02

**Authors:** Mahera Al Suqri, Huda Al-Awaisi, Mansour Al-Moundhri, Mohammed Al-Azri

**Affiliations:** 1 *Department of Psychiatry, Samail Hospital, Ministry of Health, Muscat, Oman. *; 2 *Nursing Directorate, Sultan Qaboos University Hospital, Muscat, Oman. *; 3 *Medical Oncology Unit, Department of Medicine, College of Medicine, Sultan Qaboos University, Muscat, Oman. *; 4 *Department of Family Medicine and Public Health, College of Medicine and Health Sciences, Sultan Qaboos University, Muscat, Oman. *

**Keywords:** Seeking help, delayed, cancer, colorectal, Oman

## Abstract

**Objective:**

Colorectal cancer (CRC) is the fourth leading cause of mortality in Oman, with most patients diagnosed at advanced stages. Early diagnosis of CRC improves prognosis and survival rate. The aim of this study was to explore the symptom perceptions and help-seeking behaviours (HSBs) of Omani patients diagnosed with late-stage CRC.

**Methods:**

Semi-structured individual interviews were conducted with 16 patients.

**Results:**

Four main themes emerged, including normalisation and ignorance (patients felt healthy, perceived symptoms as not being serious and related to dietary habits, concealed them or prioritised work and family commitments), self-empowerment and self-management (patients were stubborn, employed ‘wait and see’ approach, used symptomatic or herbal treatments), disclosure and seeking help (patients disclosed symptoms to family members or friends, sought medical help only when symptoms worsened, visited faith healers or travelled abroad for treatment) and healthcare professionals (patients attributed treatment or diagnosis delays to lack of continuity of care, loss of trust in doctors or delays in referral).

**Conclusion:**

Patients attributed delays in CRC diagnosis to several factors based on their perceptions of symptoms. Most HSBs driven by sociocultural and emotional causes. Increased awareness of CRC symptoms and modifying HSBs can encourage early diagnosis. Prompting patients to disclose CRC-related symptoms may aid referral decisions.

## Introduction

Colorectal cancer (CRC) is the third most common cancer worldwide after lung and breast cancer and the second leading cause of cancer-related deaths after lung cancer (Nikolouzakis et al., 2018). Moreover, the overall incidence of CRC is increasing in developing countries in comparison to developed countries, particularly among men (Rawla et al., 2019). The disease has a severe negative impact on the quality of life of affected patients and survivors for various reasons, including fatigue, sleep disturbances and urinary and sexual problems (Magaji et al., 2019). In Oman, the incidence and prevalence of CRC has increased dramatically over the past 15 years, with approximately 60% of patients presenting at delayed stages at the time of diagnosis (i.e. stages III and IV) (Al-Lawati et al., 2019). Indeed, early diagnosis of CRC improves survival rates and reduces psychological distress for affected patients and their families (Hansen et al., 2011).

A delay in diagnosis is defined as a prolonged interval between the onset of the first symptom of cancer and the start of treatment while the case navigates the diagnostic pathway from patient to primary care and then secondary care (Andersen et al., 2009; Dobson et al., 2014). Although patients are generally accepting of the idea of primary care-based cancer care services due to added convenience and minimal waiting times, research shows that delays on the part of the patients themselves are often the main reasons for delays in cancer diagnoses (McLachlan et al., 2015; Idris et al., 2020). Most delays usually occur in the help-seeking interval between the appraisal of symptoms or bodily changes and consulting a doctor (McLachlan et al., 2015). Approximately half of the patients who are diagnosed with CRC report a period of 3 months or more between symptom recognition and the first consultation with a doctor (Hamilton and Sharp, 2004). Although few frameworks are used in cancer diagnostic research, Andersen presented a general model of total patient delay (‘the Andersen model’) to account for the total time between first noticing a symptom to seeking medical help (Walter et al., 2012). 

This theoretical model of total patient delay incorporates various stages, including appraisal (time taken to evaluate a symptom as a sign of illness), illness (time taken from the first sign of illness until deciding to seek medical help), utilization (time taken from the decision to seek medical help until consulting a doctor), behavioural (time between deciding an illness requires medical help and deciding to act on this decision), scheduling (time between deciding to act on the decision to seek medical help and attending an appointment) and treatment (time between the first appointment with a doctor and the start of treatment). This model has been used in several previous studies to evaluate help-seeking behaviours (HSBs) and delays in CRC diagnosis (Walter et al., 2012; Oberoi et al., 2016). 

There is evidence that certain psychosocial factors are associated with delays in HSBs (McLachlan et al., 2015). For instance, the time taken to seek medical help is shorter among patients who perceive their symptoms to be serious and disclose them promptly to their doctors (Adelstein et al., 2012). Additionally, patients can sometimes misinterpret CRC-related symptoms as being due to non-medical causes or may present with non-specific symptoms which further delay the diagnosis (Oberoi et al., 2016). Furthermore, negative beliefs, such as fear of a cancer diagnosis and embarrassment at the idea of a physical examination, can also cause patients to delay engaging in appropriate HSBs (Oberoi et al., 2016; Al-Azri et al., 2016). On the other hand, false reassurance can lead patients to regard their symptoms as not being serious, thus contributing to delays in the decision to seek medical help or attend CRC screening programmes (Barnett et al., 2018).

In Oman, previous research has shown that most members of the public were not aware of CRC symptoms and encountered several emotional barriers to seeking early medical help (Al-Azri et al., 2019). Furthermore, Omani patients often decide to travel abroad to seek different treatment modalities and to maintain privacy due to perceptions of cancer-related stigma (Al-Azri et al., 2014). To our knowledge, no previous studies conducted in Oman have yet explored the symptom perceptions and HSBs of Omani patients diagnosed with late-stage CRC. 

## Materials and Methods

*Methods used*

Qualitative research contributes significantly to psychosocial aspects of patient care, allowing a greater understanding of these phenomena in a natural setting and examination of the experiences and views of participants in more detail (Rahman and Majumder, 2013). In our study, qualitative methods were chosen in order to help understand the attitudes and beliefs of patients and how they interpret or behave in response to symptoms as a component of their HSBs (Patton and Cochran, 2007). The data were collected via semi-structured individual interviews to gather detailed insights from patients while still ensuring a ﬂexible approach which would allow us to respond or elaborate further when faced with information that was more important than previously thought (Gill et al., 2008).

*Interviews and topic guide*

The individual interviews were conducted using a topic guide covering all potential subject areas which could arise regarding CRC-related symptom perceptions and delays in diagnosis. The topic guide was based on the five stages of delay outlined in the Andersen model of total patient delay, including the appraisal, illness, behavioural, scheduling and treatment stages (Walter et al., 2012) (see Appendix 1). In our study, the topic guide was translated into Arabic as the interviews themselves took place in this language. Semi-structured individual interviews were conducted by the first author with each patient, with the location depending on the reason for their visit to the hospital. The first author is a psychiatrist resident doctor with background experience in psychology, counselling and establishing a rapport with patients, all of which are essential components of qualitative interviewing (Dicicco-Bloom and Crabtree, 2006). Each interview lasted between 30–45 minutes and was audio-recorded and transcribed verbatim. 

*Recruitment of participants*

Sultan Qaboos University Hospital (SQUH) is a tertiary teaching and training hospital located in Muscat, the capital of Oman. SQUH provides comprehensive oncological treatment to patients diagnosed with cancer and referred from other governmental or private hospitals from other regions of Oman. A convenience sampling strategy was used to collect information because the subjects were readily available at any given time and easily accessible to the researcher (Etikan et al., 2016). 

All Omani patients who had been diagnosed with CRC over the preceding six months were identified from the electronic hospital information system. Subsequently, only patients diagnosed with advanced stages of CRC based on TNM staging were included (Weiser, 2018). The date of their next appointment to the oncology wards, outpatient oncology clinic or day care for follow-up, admission or treatment was recorded. Identified patients were contacted and the purpose of the study was explained to them. All potential participants were assured regarding the confidentiality and anonymity of collected information. Patients were informed that participation in the study was optional and would not affect the medical care they received. 

Only patients who agreed to participate and who attended the outpatient oncology clinics or day care units or those who were admitted to the wards during the study period were included. Patients who were in obvious distress or pain were excluded and another patient was contacted in their place. All participants signed a consent form prior to taking part in the study. 

*Data analysis*

The framework analysis method was utilised to analyse the collected data. This approach was chosen as it ensures flexibility during the analysis and can be used when data collection and analysis occur concurrently (Ritchie and Spencer, 1994). The framework analysis involved five steps, including (1) process stage familiarization, (2) identifying a thematic framework, (3) indexing, (4) charting and (5) mapping and interpretation. Initially, the researchers became familiar with the interview techniques and equipment, including the processes of audio-recording and transcription. Subsequently, coding was used to identify a thematic framework developed both from a priori and emerging issues discovered during the familiarisation stage. A deductive coding approach was applied as the codes were pre-defined based on the five key stages outlined in the Andersen model of total patient delay (Walter et al., 2012; Palinkas et al., 2015). The third step was indexing whereby the thematic framework was applied to the data using numerical or textual codes to differentiate specific themes. The fourth step involved charting wherein headings from the thematic framework were used to create charts to summarise the data according to specific categories from each transcript for each theme. Finally, the mapping and interpretation stage constituted defining specific concepts, searching for patterns and identifying associations (Srivastava and Thomson, 2009). 

The first, second and last authors independently coded the initial interview transcripts, with all authors meeting in order to reach a consensus on the final set of codes to be applied to all subsequent transcripts. Emergent themes and subthemes were then discussed with the other authors, all of whom are healthcare professionals with experience in qualitative research. The data collection process was halted once the saturation point in the analysis had been achieved (i.e. no new codes were identified during data analysis and no new information could be elicited from the participants) (Patton, 2002).

## Results

*Characteristics of the study participants*

A total of 16 participants diagnosed with late-stage CRC were interviewed. Of these, nine were male and seven were female. The mean age of the participants was 53 years (median: 52 years; range: 36 to 72 years). The majority of participants were diagnosed with stage IV CRC (n = 9). The reported time between the initial onset of possible CRC-related symptoms to the first consultation with a doctor ranged from two weeks to 26 months, with a mean of nine months between the first onset of possible symptoms and the time of diagnosis (see Table 1).

*Main themes*

We identiﬁed four main themes to describe the symptom perceptions, experiences and HSBs of participants which could have contributed to delays in CRC diagnosis. The four main themes covering the decision-making processes, behaviours and responses adopted by the participants were: normalisation and ignorance; self-empowerment and self-management; disclosure and seeking help; and healthcare professionals and health care system-related factors. Examples of specific quotes that illustrate and support these themes are presented in Table 2.

*Normalisation and ignorance*

Participants reflected on bodily changes that were subsequently recognised as CRC-related symptoms. Several participants mentioned that they felt healthy and believed that certain symptoms such as constipation or diarrhoea were not serious and therefore did not warrant medical attention, particularly as they did not disturb their daily activities or functioning (see Table 2, quotes 1 and 2).

Moreover, intermittent or mild symptoms such as abdominal pain and unintentionally losing weight were often ignored until they became more severe (see Table 2, quotes 3 to 5). Several participants ignored symptoms despite advice from family members or relatives to seek medical help (see Table 2, quotes 4 and 5). One participant intentionally concealed their symptoms from family members, claiming that he was never asked (see Table 2, quote 6). Other individuals ignored symptoms as a result of prioritising family or work commitments (see Table 2, quotes 7 and 8).

In addition, participants often dismissed their symptoms as a result of misconceptions. Some participants misinterpreted CRC-related symptoms (i.e. diarrhoea and the passing of blood in the stool) as being related to dietary habits, such as drinking ginger to treat high cholesterol or eating black pepper (see Table 2, quotes 9 and 10). Others mistakenly believed that CRC only occurred in men and not women and therefore attributed symptoms to organs other than the colon. For example, a female participant had symptoms of bloating and lower abdominal pain which persisted for two years, but consulted her gynaecologist for suspected uterine cancer as she thought CRC could only occur in men (see Table 2, quote 11).

*Self-empowerment and self-management*

Self-management and self-empowerment was a prominent theme mentioned by several participants. Such participants often displayed stubbornness or a belief that they should act ‘strong’ before seeking medical help or telling their family members of their symptoms, especially as they thought that the symptoms would resolve eventually. In particular, the participants perceived that telling spouses or relatives of their symptoms or seeking medical help might restrict their activities or make them look weak in front of others (see Table 2, quotes 12 and 13). Other participants applied a ‘wait and see’ approach in the similar hope that symptoms would disappear, which often resulted in prolonged delays before seeking medical help (see Table 2, quotes 14 and 15).

Other subthemes in this category included the use of traditional herbal medicine or over-the-counter remedies as forms of symptomatic treatment. Several participants used traditional herbal treatments, such as karpooravalli (Indian borage), anise, ginger and olive oil (see Table 2, quotes 16 and 17). Some also took over-the-counter medications to relieve symptoms of constipation, bought vitamin supplements to manage weight loss or tiredness and utilised a sitz (hip) bath to soothe what they believed to be haemorrhoid pain (see Table 2, quotes 18 and 19).

*Disclosure and seeking help*

The third theme identified was disclosure and seeking help. This change in HSBs could reflect the transition from ignorance or denial to a perception of something being abnormal, especially when symptoms worsened, became more severe or persistent, when attempts at self-medication failed, if there was a sudden onset of new symptoms or the bodily changes began to interfere with daily activities. Some participants disclosed their symptoms to family members, friends or colleagues first before seeking medical help, perhaps for the purposes of normalisation or reassurance. For example, a female participant disclosed her symptoms to her colleagues and was incorrectly told that she had haemorrhoids (see Table 2, quote 20). Another participant discussed her symptoms with her family members only when the symptoms worsened (see Table 2, quote 21).

Several participants decided to seek help from faith healers to receive a spiritual explanation for their symptoms. These participants were often informed that the cause of their symptoms was due to envy or the ‘evil eye’ (see Table 2, quotes 22 and 23). Other participants who had previously ignored bodily changes sought medical help, but only when their symptoms worsened, they developed additional symptoms or complications or when the symptoms began to impair their normal daily activities (see Table 2, quotes 24 and 25).

Participants also reported travelling abroad (e.g. to Thailand or India) for medical help for a variety of reasons, including seeking a secondary opinion, to undergo investigations and treatments either before or after receiving a confirmed diagnosis of CRC or due to long waiting times for referral to a specialist (see Table 2, quotes 26 and 27). Some participants also reported being pressured by their family members to travel abroad for treatment (see Table 2, quotes 28 and 29).

*Healthcare professionals and healthcare system-related factors*

The final theme incorporated various attitudes towards and perceptions of healthcare professionals and the healthcare system. One subtheme was the lack of continuity of care, in which some participants underwent consultations with several different doctors and therefore received conflicting opinions resulting in confusion (see Table 2, quote 30). Other participants reported that they became frustrated by having to attend so many appointments without any investigations or lost faith in their doctors after receiving false reassurance (see Table 2, quotes 31 and 32). In several cases, participants reported delays in referral either due to a lack of timely appointments or only being referred after several visits or when their symptoms had worsened or complications had developed (see Table 2, quotes 33 to 35). 

**Table 1:** Characteristics of the Study Participants and Medical Stage at Diagnosis (n = 16).

Characteristic	n (%)
Gender	
Male	9 (56.3%)
Female	7 (44.8%)
Age (years)	
Minimum	36
Maximum	72
Mean	53 (SD = 11.34)
Median	52 (SD = 11.34)
Educational level	
No formal education	6 (37.5%)
Primary	3 (18.8%)
Secondary	3 (18.8%)
University and postgraduate	4 (25.0%)
Region of origin	
Muscat governorate	5 (31.3%)
North Al-Batinah	4 (25.0%)
South Ash Sharqiyah	3 (18.6%)
South Al-Batinah	2 (12.5%)
Ad Dakhiliyah	1 (6.3%)
Dhofar	1 (6.3%)
Type of cancer	
Colon	14 (87.5%)
Rectal	2 (12.5%)
Stage at diagnosis (TNM)	
IIA (The cancer has grown through the wall of the colon or rectum)	1 (6.3%)
IIB (The cancer has grown through the visceral peritoneum)	3 (18.8%)
IIIB (The cancer has grown through the bowel wall or to surrounding or gans and into 1 to 3 lymph nodes)	3 (18.8%)
IV (The cancer has spread to distant parts of the body)	9 (56.3%)
Mean reported time from first symptom to seeking medical help (months)	9 (SD = 8.19)
Presence of co-morbidities	
Diabetes mellitus	3 (18.8%)
Hypertension	2 (12.5%)
Dyslipidaemia	2 (12.5%)
Hypertension and dyslipidaemia	2 (12.5%)
Diabetes mellitus and hypertension	2 (12.5%)
None	5 (31.3%)

SD, Standard Deviation; T, Tumor; N, Node; M, Metastasis

**Table 2:** Summary of Themes and Supporting Quotes from the Study Patients (n = 16).

Main theme	Type of HSB/ action	Supporting quote
Normalisation and ignorance	Feeling healthy or normal	Q1. “Yes... Sometime I get a mild diarrhoea and sometimes I am mildly constipated. It’s not like I get severe diarrhoea or constipation. It’s not like I don’t have bowel movements at all. I mean, to me, I consider it normal… I never thought of it as anything abnormal… all normal.” (P12: *50-year-old woman with stage IV colon cancer*). Q2. “I have had chronic constipation since a long time… and, to me, it was normal… That never bothered me.” (P16: *43-year-old woman with stage IIIB colon cancer).*
	Ignorance/denial	Q3. “My first symptoms started showing last September. Started as daily stomach ache most of the times... After September, I was getting these episodes more frequently, very painful… I only started looking into the issue in April, about eight months later… I neglected the issue and looked into it later.” (P3: *43-year-old woman with stage IIIB colon cancer*).
	Ignorance/denial, despite advice from family members or relatives	Q4. “When I first experienced these things [symptoms], I did not feel anything, even my children were telling me that I have lost weight… My family were asking me to go for a check-up, but I neglected myself.” (P11: *70-year-old man with stage II colon cancer)*. Q5. “In the beginning, I only told my husband. Then I told my mother when my pain and cramps became more severe... I told my sister who is a nurse… she felt that it could be serious and that what I am going through is not normal and asked me to go to the hospital immediately. I neglected the issue. I did not think that I may end up having a tumour.” (P16: *43-year-old woman with stage IIIB colon cancer*).
	Concealing symptoms	Q6. “When I got the pain, I did not tell them, because no one asked. But I have these fears and thoughts, just like obsessions in my mind.” (P9: *39-year-old man with stage IV colon cancer*).
	Prioritising family/work commitments	Q7. “We are Bedouin tribe who live in the desert… since early morning, we head to the farm and then to buying fish from the fish market and so on…. We don’t feel anything.” (P14: *72-year-old man with stage IIIB colon cancer*). Q8. “My wife is sick and I am busy with her treatment. I’ve been to Thailand and India for her treatment, all the while having constipation.” (P10: *43-year-old man with stage IV colon cancer*).
	Dismissing symptoms as related to dietary habits	Q9. “Lately, I would sometimes get diarrhoea… but I thought the reason I was getting it was because I drink a cup of warm ginger every morning… I had no pain... it was just diarrhoea four to five times daily… I thought it’s normal, especially since it wasn’t affecting me.” (P5: *51-year-old man with stage IIIB colon cancer)*. Q10. “I think it is because of black pepper… I noticed I had blood with my stool bright red… I thought it was because of black pepper and these things.” (P8:* 65-year-old man with stage IV rectal cancer*).
	Dismissing symptoms due to gender misconceptions	Q11. “They always tell us that, for women, cancer is either breast cancer or uterine cancer... It is like I was blind and I couldn’t see, all I thought was that colon cancer happens only in men… I did not think it might happen to women too.” (P12: *50-year-old woman with stage IV colon cancer*).
Self-empowerment and self-management	Being stubborn	Q12. “I was stubborn with my illness. I told myself, I will resist the illness with any means possible… Maybe that way it will change or even go away. That was the principle… I like to be active, I never thought that one day I might be admitted to a hospital.” (P1: *59-year-old man with stage IV colon cancer*). Q13. “My husband was telling me over and over again to go and get checked. I am a stubborn person, I told you that before. I did not think the situation would reach to this extent.” (P16: 4*3-year-old woman with stage IIIB colon cancer*).
	Adopting a ‘wait and see’ attitude	Q14. “I am a patient person, I thought maybe it is a simple thing, that if I wait, it will go. Every person has his way in life, and this is my way, I wait.” (P5: *51-year-old man with stage IIIB colon cancer*). Q15. “I have constipation and I had pain around the anus... and also while defecating. I thought maybe it will go.” (P3:* 43-year-old woman with stage IIIB colon cancer*).
	Using herbal medicine	Q16. “I have constipation for sure... and for a month I was using traditional herbal treatments for constipation.” (P14:* 72-year-old man with stage IIIB colon cancer*). Q17. “For a month, I would probably have severe constipation for three to four days, I’d hardly be able to pass stool. I never saw a doctor. I would treat myself with traditional remedies, mainly drinking anise and other herbs.” (P3: *43-year-old woman with stage IIIB colon cancer*).
	Using over-the-counter and symptomatic treatments	Q18. “I mean, I would go get stuff from the pharmacy... or tell them I want my weight to increase and they would give me vitamins and other supplements, which I took but not regularly.” (P1: *59-year-old man with stage IV colon cancer)*. Q19. “It was like I had numbness or pain in the area between my hips from the back... it was like numbness. I would put cumin in hot water, put it in a sitz bath and sit in it. Once the area is warm enough, I’d be able to sleep with no pain.” (P6: *60-year-old woman with stage IIIB rectal cancer*).
	Type of HSB/ action	Supporting quote
	Disclosing symptoms to family members, colleagues or friends when symptoms worsened or became painful	Q20. “I asked my colleagues and co-workers. I told them I had pain just like labour pain, but from behind, as in anally… they were telling what I had was haemorrhoids.” (P3: 43-year-old woman with stage IIIB colon cancer). Q21. “For a week, I’d be eating normally and still would not pass stool... very hard stool... I stayed like that for a while and then I told my kids... I am tired of the constipation... tired of the stomach ache... So they took me to the doctor.” (P13: 54-year-old woman with stage IV colon cancer).
	Seeking out faith healers for a spiritual explanation	Q22. “Oh my God, my stomach ache was very severe. Day and night, I wouldn’t be able to get any sleep... I was told that my body condition keeps on deteriorating and that maybe I was jinxed… Everyone said something, you know our society… I spent an entire month visiting several Sheikhs.” (P4: 45-year-old woman with stage IV colon cancer). Q23. “Even mentioned in the Quran... Issues related to envy (hassad) and stuff like that are mentioned in the Quran. He would tell you whether you have been harmed or not. The healer told me that I have been harmed.” (P10: 43-year-old man with stage IV colon cancer).
	Seeking out medical help once symptoms worsened	Q24. “Because my stomach was bloated, and I kept on being bloated and gassy. And you know, being gassy and the smell was very bad. I started not going out a lot with people just because of this. And then I went to the hospital to get checked.” (P10: 43-year-old man with stage IV colon cancer). Q25. “Yes, I am staying at my sister’s place for a week... because of the pain, I could not go back to my hometown... It got really bad, so my family brought me to emergency department.” (P4: 42-year-ld woman with stage IV colon cancer).
	Travelling abroad to seek a secondary opinion	Q26. “Once they give me a diagnosis, I’ll be sure that, yes... I was planning on getting the treatment here… So I went to Thailand and got tested… I thought maybe they are more evolved or progressive in medicine.” (P2: 36-year-old man with stage IV colon cancer).
	Travelling abroad due to long appointment times	Q27. “Here, they gave me an appointment for colonoscopy, but I didn’t wait for it because it was after a month… and I could not wait with all the pain I had, so I decided to go abroad.” (P3: 43-year-old woman with stage IIIB colon cancer).
	Travelling abroad due to pressure from family members	Q28. “My family urged me to travel... they told me from the beginning: go to a specialised hospital abroad.” (P5: 51-year-old man with stage IV colon cancer). Q29. “It was my family’s decision… I don’t have an issue getting my treatment here or abroad, but the family got together and decided that I go to India for treatment.” (P15: 53-year-old woman with stage IV colon cancer).
	Confusion regarding conflicting opinions	Q30. “I don’t know... many follow-ups... some tell you one thing and the others tell you something else... They don’t give you one opinion.” (P4: 42-year-old woman with stage IV colon cancer).
	Frustration due to lack of progress	Q31. “I had enough... I don’t want to go…. Every day, coming from my town… appointments... appointments every day. You get bored of so many appointments. I go to check up on my blood pressure and, when am by the hospital door, my blood pressure shoots up and I become annoyed, you know.” (P6: 60-year-old woman with stage IIIB rectal cancer).
	Loss of trust with the doctors	Q32. “Yes. It’s like you are reassuring me that everything is normal, colonoscopy results are normal, I don’t need tablets, and everything is normal… And then, in an instant, you come and tell me, now you need surgery… I mean, I... I don’t trust you.” (P9: 39-year-old man with stage IV colon cancer).
	Lack of timely appointments	Q33. “Yes, whenever I would ask for an earlier appointment… They would say there isn’t any appointment available. They said there are a lot of people, you have to wait your turn... and, like that, they made me wait two months at home.” (P8: 65-year-old man with stage IV rectal cancer).
	Referred only after several visits or after symptoms worsened or following complications	Q34. “Constipation would get worse... the pain would get worse... My kids took me to the health centre, he [the doctor] gave me pills and told me to go back home… Then the pain worsened and I started throwing up... I couldn’t even breathe… When I got back to them for the third time, they admitted me and then they transferred me to hospital and admitted.” (P13: 54-year-old woman with stage IV colon cancer).
		Q35. “The bloating increased, and the pain increased... I went to the polyclinic and they did X-rays and blood tests... You have to go to the one hospital… I went to... They admitted me for around four hours, then gave me an appointment after five months... I was throwing up at home and tossing and turning... And then, just to try our luck, my son took me to another hospital… There, they did an endoscopy and doctor told me that I needed surgery.” (P11: 70-year-old man with stage II colon cancer).

## Discussion

To our knowledge, this is the first study conducted in Oman to explore the symptom perceptions and HSBs of Omani patients diagnosed with late-stage CRC. Approximately half of our participants experienced delays in presentation of up to 28 months. The usual interval between the onset of CRC-related symptoms and seeking medical attention ranges from three to six months (Esteva et al., 2013). Failure on the part of our participants to perceive the seriousness of their symptoms and thus seek early medical attention might be related to a lack of knowledge of CRC-related symptoms. Indeed, many of our participants misinterpreted their symptoms as not serious enough to warrant medical attention, particularly if they did not interfere with daily life, or incorrectly attributed them to their dietary habits or to diseases other than CRC due to their gender.

 A previous study conducted in Oman showed that overall recognition of CRC-related symptoms among members of the public was low (Al-Azri et al., 2019). Lack of awareness of cancer symptoms causes a delay in seeking medical help as people are unable to interpret their complaints as signs of cancer (Al-Azri et al., 2015). Although, there are as yet no national screening programmes for CRC in Oman, increasing knowledge may reduce negative public perceptions of cancer which may impact positively on intentions to ask for a colonoscopy referral if such symptoms subsequently develop. (McCaffery et al., 2003). Indeed, many of our participants waited to disclose their symptoms or seek medical help only once the pain became severe. Cancer patients who do not detect symptoms during the symptom appraisal phase or who hold beliefs that the absence of severe pain does not warrant any cause for concern often experience delays in cancer diagnosis (King-Okoye et al., 2017).

Socioeconomic status, educational level and emotional barriers involving cancer diagnosis (such as being worried over what the doctor might find or feeling embarrassed at the idea of a physical examination or colonoscopy) are factors which can contribute to delays in diagnosis in Oman (Al-Azri et al., 2016). On the other hand, when our participants eventually perceived their symptoms to be serious or after such symptoms had increased in intensity or began to interfere with daily life, they sought help or disclosed their symptoms to family members. Furthermore, other participants exhibited certain behaviours or attitudes in response to CRC-related symptoms, including denial, being stubborn or employing a ‘wait and see’ approach.

Although beliefs are socially constructed by individuals in relation to their environment and culture, a disturbance in the body or bodily changes can trigger beliefs which cause the individuals to interpret these changes either as normal or something that requires medical attention (King-Okoye et al., 2017). Nonetheless, while denial is an ineffective and passive defence mechanism, cancer patients often fall back on this coping strategy as a form of protection against overwhelming feelings of stress, particularly in the initial phases of a cancer diagnosis (Al-Azri et al., 2014).

Some patients in Oman are reluctant to share news of a cancer diagnosis with family members or friends in order to avoid cancer-related stigma (Al-Azri et al., 2014). On the other hand, several participants in our study disclosed their cancer symptoms to family members or friends, perhaps for emotional relief, to facilitate HSBs or to receive advice concerning treatment. The prevailing social culture in Oman is based on a strong sense of moral responsibility and familial obligation which often contrasts with a patient-centred approach to treatment decision-making, particularly in cancer cases (Al-Bahri et al., 2019). However, the involvement of family members or friends in HSBs or treatment decision-making can delay presentation as a result of false reassurances or pressure to turn to non-conventional treatment modalities (Al-Bahri et al., 2018). Indeed, many participants reported that their family members were engaged in treatment decision-making and compelled them to travel abroad for treatment; these factors might have an adverse effect including delays in cancer diagnosis (Burney, 2009).

The use of herbal remedies in the treatment of CRC-related symptoms was noted among several of our participants. These findings support those of a previous study conducted in seven Western countries in which around 32% of patients diagnosed with CRC turned to complementary therapies after diagnosis (Molassiotis et al., 2005). However, the direct impact of culture on the perception of symptoms and HSBs was apparent as many of our participants sought out faith healers for a spiritual explanation of their symptoms. Seeking help from faith healers for medical issues is still an existing practice in many Muslim countries, particularly for emotional and spiritual support (Chui et al., 2014). Indeed, cancer patients in Oman have reported several devastating psychological symptoms, such as fear of death, which may explain why many pursue traditional or spiritual remedies (Al-Azri et al., 2014). In addition, patients who use traditional medical approaches in Oman such as Wasam (cautery) often present late with advanced cancer and complications like burns and wound infections (Al-Lawati et al., 2016). 

Several participants in our study reported that they received conflicting medical advice after consulting with different doctors; moreover, some of them received false reassurance which caused them to lose trust in their doctors and stop attending appointments, all of which served to delay their CRC diagnosis (Langenbach et al., 2003). As in other Arab countries, the healthcare system in Oman does not support continuity of care with a particular doctor, thus increasing the likelihood of patients receiving conflicting opinions. Continuity of care has been found to increase the early detection of cancer and the likelihood of cancer testing; in contrast, lack of continuity increases confusion and uncertainty for the patient, thereby delaying diagnosis (Nazareth et al., 2008; Al-Azri, 2016). 

Several patients reported delays in being referred to a specialist; this factor has been found to contribute to delays in cancer diagnosis (Al-Azri, 2016). A diagnosis of cancer is relatively uncommon in primary healthcare settings as the majority of patients present with non-specific symptoms (Green et al., 2015). Thus, physicians may concentrate on symptomatic management without referring the patient to a specialist for a colonoscopy (Green et al., 2015; Al-Azri, 2016). Furthermore, some cancer patients do not attend referral appointments due to emotional barriers or because they prioritise work or family commitments, further delaying the diagnosis (Al-Azri et al., 2015; Oberoi et al., 2016; Al-Azri et al., 2016;). Indeed, raising awareness of public for the importance of screening such as faecal occult blood (FOB) or colonoscopy are proven to enhance early diagnosis and be effective in preventing CRC-related deaths (Zhang et al., 2017).

*Limitations*

This study is the first study conducted in Oman to explore the symptom perceptions and HSBs of Omani patients diagnosed with late-stage CRC. Although generalisability is not usually expected in qualitative research, participants were recruited from a single site and thus our findings may not be applicable to individuals living in other countries with different cultures or with alternative healthcare systems. Furthermore, our study might have been more beneficial if healthcare providers had been involved so that we could obtain and compare information regarding reasons for delays in diagnosis from their perspective.

In conclusion, the participants’ perceptions of CRC symptoms were driven by sociocultural and emotional factors; moreover, HSBs were found to delay CRC diagnosis during the help-seeking process. Whilst there is a need to respect the individual beliefs, cultures and perceptions of all patients, understanding how these factors impact how patients appraise symptoms and seek help is important to help minimise delays in diagnosis. Healthcare professionals should encourage patients to disclose additional information during consultations, particularly in the context of potential cancer symptoms, and acknowledge their responses without conferring shame or blame. 

Although HSBs are influenced by beliefs and culture which might alter depending on society and previous experiences, implementing a continuity of care system may help to promote timely HSBs by avoiding confusion and increasing the patient’s trust in their doctor, both of which could ensure earlier CRC diagnoses. Furthermore, strengthening this relationship could shed light on and correct other beliefs that underpin HSBs which counteract against early medical help-seeking. In particular, prompting patients for more CRC-related symptoms could aid early referral decisions.

Our findings mandate increased efforts to raise awareness of CRC-related symptoms in Oman and to develop interventions that encourage individuals to seek timely medical advice. Nonetheless, correctly recognizing and interpreting CRC-related symptoms is only possible if this knowledge influences beliefs about symptom experience and, in turn, HSBs. Indeed, it may be valuable to consider a more inclusive approach and target these issues with traditional and social media campaigns, for instance by distributing informational posters and leaflets in healthcare institutes to increase awareness of CRC-related symptoms, and by implementing screening. Finally, the current study showed that family played an important cultural role in symptom appraisal and may be a potential source of help in influencing HSBs. Thus, doctors should be aware of their influence when patients present with CRC-related symptoms; attempts to counteract early medical help-seeking or make use of alternative treatments should be modified and corrected. 
